# Brain imaging signatures of neuropathic facial pain derived by artificial intelligence

**DOI:** 10.1038/s41598-023-37034-y

**Published:** 2023-07-03

**Authors:** Timur H. Latypov, Matthew C. So, Peter Shih-Ping Hung, Pascale Tsai, Matthew R. Walker, Sarasa Tohyama, Marina Tawfik, Frank Rudzicz, Mojgan Hodaie

**Affiliations:** 1 Division of Brain, Imaging & Behaviour, Krembil Research Institute, University Health Network, Toronto, ON Canada; 2 Institute of Medical Science, Temerty Faculty of Medicine, University of Toronto, Toronto, ON Canada; 3 Collaborative Program in Neuroscience, University of Toronto, Toronto, ON Canada; 4 Max Rady College of Medicine, University of Manitoba, Winnipeg, MB Canada; 5 Michael G. DeGroote School of Medicine, McMaster University, Hamilton, ON Canada; 6 A.A. Martinos Center for Biomedical Imaging, Harvard Medical School, Charlestown, MA USA; 7 Department of Computer Science, University of Toronto, Toronto, ON Canada; 8 Vector Institute for Artificial Intelligence, Toronto, ON Canada; 9 Faculty of Computer Science, Dalhousie University, Halifax, NS Canada; 10 Division of Neurosurgery, Department of Surgery, University of Toronto, Toronto, ON Canada

**Keywords:** Neuropathic pain, Neurological disorders

## Abstract

Advances in neuroimaging have permitted the non-invasive examination of the human brain in pain. However, a persisting challenge is in the objective differentiation of neuropathic facial pain subtypes, as diagnosis is based on patients’ symptom descriptions. We use artificial intelligence (AI) models with neuroimaging data to distinguish subtypes of neuropathic facial pain and differentiate them from healthy controls. We conducted a retrospective analysis of diffusion tensor and T1-weighted imaging data using random forest and logistic regression AI models on 371 adults with trigeminal pain (265 classical trigeminal neuralgia (CTN), 106 trigeminal neuropathic pain (TNP)) and 108 healthy controls (HC). These models distinguished CTN from HC with up to 95% accuracy, and TNP from HC with up to 91% accuracy. Both classifiers identified gray and white matter-based predictive metrics (gray matter thickness, surface area, and volume; white matter diffusivity metrics) that significantly differed across groups. Classification of TNP and CTN did not show significant accuracy (51%) but highlighted two structures that differed between pain groups—the insula and orbitofrontal cortex. Our work demonstrates that AI models with brain imaging data alone can differentiate neuropathic facial pain subtypes from healthy data and identify regional structural indicates of pain.

## Introduction

Chronic pain conditions affect up to 20% of the population and are among the most common presenting complaints from adults seeking medical care^1–3^. An ongoing challenge for the clinical and research communities is the lack of objective assessment and diagnostic criteria for these conditions^3,4^. For example, trigeminal neuralgia (TN) is a common neuropathic facial pain that is frequently referred to neurosurgical care, yet its diagnosis can be complex. TN is often diagnosed in the context of neurovascular compression of the trigeminal nerve, however, neurovascular compression does not pre-define pain in TN. In fact, up to 40% of patients diagnosed with TN may not demonstrate this type of vascular compression on MR imaging^5^. Furthermore, lancinating pain is a common descriptive symptom for trigeminal pain syndromes such as classical trigeminal neuralgia (CTN), persistent idiopathic facial pain, and secondary trigeminal pain syndromes^6–8^. However, these syndromes may have very different etiologies^9^. The limited precision of clinical diagnosis of pain may result in patients receiving a treatment plan that does not specifically align with their form of chronic facial pain and provides limited relief^3,10–12^.

Extensive studies using functional and structural brain imaging have uncovered objective neuroanatomical signatures of chronic facial pain and consistently point to gray matter volume and thickness abnormalities, including at the insula, anterior cingulate cortex, thalamus, and hippocampus^13–15^. Yet, the impact of these candidate biomarkers remains poorly discerned, and not integrated within clinical practice due to the lack of generalizability and reproducibility^16,17^. Another factor that hinders the clinical utility of these studies is their incorporation of conventional statistical approaches to delineate structural differences, as the multiple tests problem makes classical statistical interference less tractable^18^. The use of advanced analysis methods such as artificial intelligence (AI) tools offer a more effective way of analyzing large datasets^18–20^. AI tools have been increasingly sought due to their ability to identify undiscovered patterns that connect complex and heterogeneous forms of neurological disease^21–24^. For example, neuroimaging datasets with diffusion tensor (DTI) and T1-weighted imaging information typically contain large numbers of features that cannot be easily analyzed using conventional statistical approaches^23,25,26^. The inherent complexity of these imaging datasets is highly suitable for AI models, as they can efficiently analyze unstructured data where the number of input variables exceeds the number of subjects^18,19^.

Multiple forms of AI tools, including unsupervised and supervised learning models, can be used to guide data-driven analyses. Unsupervised learning can identify hidden patterns of unlabeled data while supervised learning can precisely and consistently handle the labelled data, such as predicting the outcome of the surgical intervention, or identifying the diagnosis^24,27,28^. Both unsupervised and supervised learning AI techniques benefit from large sample sizes (*n* > 100) and can reveal new foci which may not be elucidated in traditional hypothesis-driven investigations^24,28^. Importantly, AI techniques frequently use multimodal data, including combining brain imaging with clinical charts data. Since retrospective clinical chart data points are inherently subjective, they can potentially introduce bias and decrease the accuracy of the model^29,30^. This moves us to inquire whether imaging data alone is sufficiently able to predict pain from non-pain states. If so, are AI tools capable of distinguishing between different types of chronic facial neuropathic pain^24^.

In the present study, we conduct a retrospective AI-driven analysis of brain imaging (DTI, T1-weighted imaging data) from chronic facial pain patients diagnosed with either CTN or trigeminal neuropathic pain (TNP) and healthy controls. We inquire whether unsupervised and supervised learning AI tools may be sufficiently powerful to distinguish chronic facial pain from healthy controls based on brain imaging data alone, and what are the neuroanatomical signatures that distinguish chronic facial pain subtypes from healthy controls. We hypothesized that unsupervised and supervised learning AI tools applied to neuroimaging data can distinguish CTN and TNP subtypes of chronic facial pain from healthy controls, and that AI tools can identify neuroimaging signatures that distinguish CTN and TNP from healthy control (HC) data.

## Results

### Subject demographics

We used data from 371 adults with chronic facial pain conditions and 108 HC. Facial pain subjects were divided into two groups (CTN and TNP) according to their clinical diagnoses. We identified 265 adults with CTN, and 106 with TNP. The TNP group included patients with idiopathic and secondary symptomatic variants of trigeminal pain (idiopathic facial pain, deafferentation pain and postherpetic neuralgia). No statistically significant differences were found in mean age, duration of pain, pain intensity nor the proportion of left/right-sided pain or proportion of males/females across populations. Complete demographic information and diagnosis codes are shown in Table 1.

**Table 1:** Group numbers and mean ± SD of time-based factors.

	CTN	TNP	HC
ICHD-3 codes	13.1.1.1	13.1.2.2; 13.1.2.3; 13.1.2.5	NA
N	265	106	108
L/R pain side (proportion)	115/150 (0.43/0.57)	43/63 (0.43/0.57)	–
Male/female (proportion)	95/170 (0.36/0.64)	37/69 (0.35/0.65)	47/61 (0.45/0.55)
Mean age at a time of follow-up in years (SD)	59.1 ± 12.6	56.5 ± 15.7	55.6 ± 16.2
Mean duration of pain in years (SD)	8.3 ± 7.5	7 ± 6.5	0
Type of pain	Episodic	Constant	No pain
Pain intensity	8.1 ± 2.4	8.7 ± 1.8	0

*CTN* classical trigeminal neuralgia, *TNP* trigeminal neuropathic pain, *HC* healthy controls.

### Healthy control datasets

In order to confirm the usability of healthy control data from the CamCAN dataset and our internal collection, we conducted two-one-sided t-tests (TOST)^31^ on imaging data from age- and sex-matched subjects (n = 62 from each dataset) between these two sets. The TOST procedure showed significant *p*-values for all regions (*p* < 0.001), rejecting the null hypothesis of TOST (Supplemental Fig. 2, Supplemental Table 2).

In addition, the t-distributed stochastic neighbour embedding visualization of data uncorrected for the pain side showed no specific clustering with regard to the data source (supplemental Fig. 3). Given the similarity of the distributions of two datasets confirmed by two different methods, we conclude that these datasets are suitable for combined AI-driven analyses.

### Unsupervised learning and data visualization

**Figure 1:**
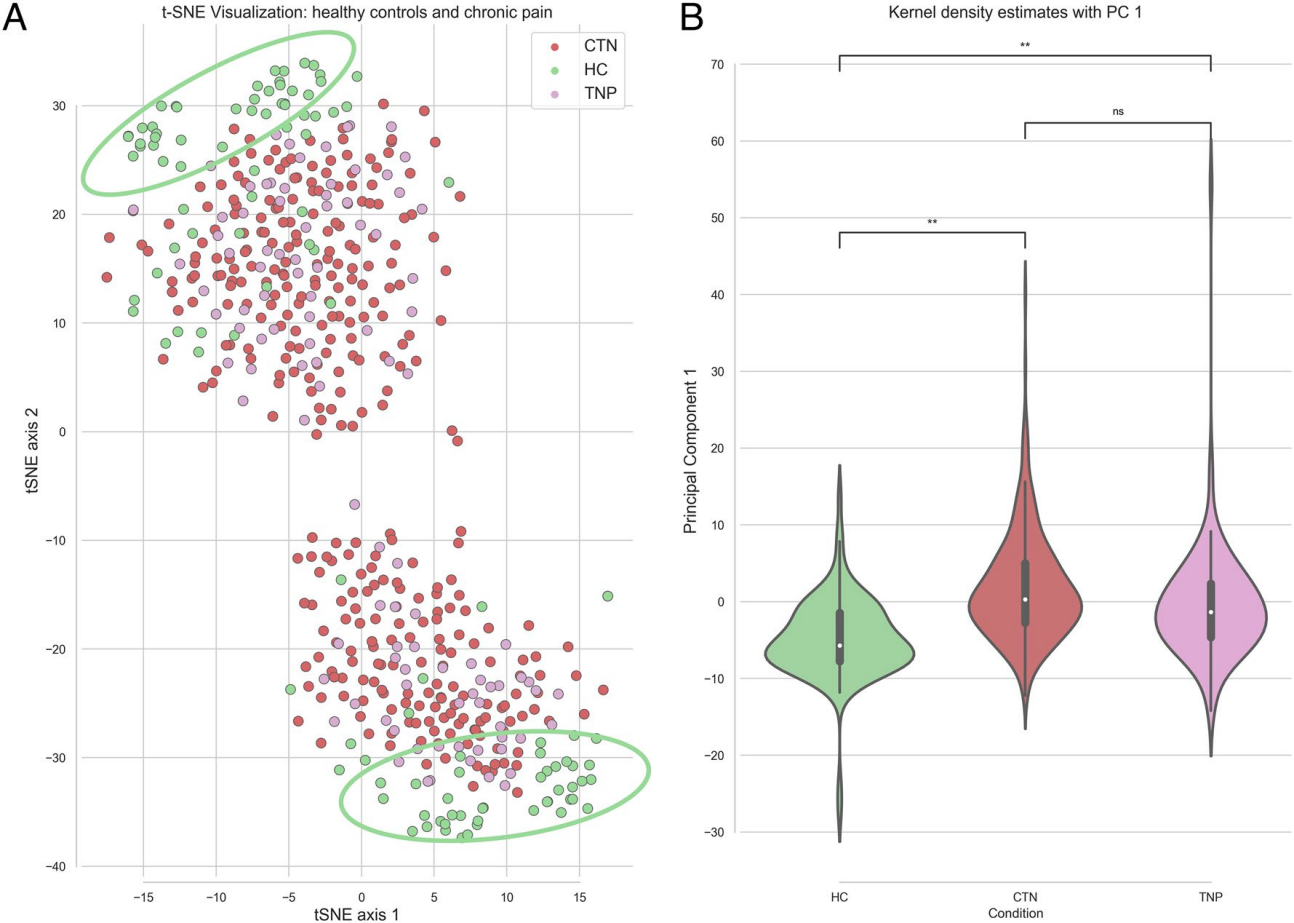
Unsupervised learning and data visualization using t-SNE (**A**) and PCA (**B**) demonstrates the separation of healthy individuals (green) from chronic facial pain subjects (red and pink). The t-SNE axes on the plot are arbitrary. PC1 axis represents all variables of the dataset in one dimension. *T-SNE* t-distributed stochastic neighbour embedding, *PCA* principal component analysis, *PC1* Principal components axis 1.

We used the principal component analysis (PCA) and t-distributed stochastic neighbour embedding (t-SNE) dimensionality reduction unsupervised learning approaches to visualize our multivariate data. The t-SNE analysis revealed two major clusters, with the primary factor contributing to the separation being the hemisphere affected by TN, along with the corresponding assignment of the ipsilateral hemisphere on the healthy controls’ data. Within each cluster, the separation of healthy controls from subjects with trigeminal pain was observed. Comparison of data uncorrected for the pain laterality showed a similar trend within one large cluster (Supplemental Fig. 3). Both t-SNE and PCA methods were able to differentiate between pain and HC groups. The differentiation was statistically significant in PC1 (*p* < 0.01). Visualization in PC1 and PC2 did not result in a clear separation of different groups (Supplemental Fig. 4). CTN and TNP appeared relatively similar on both t-SNE visualization of the T1 and DTI scalar data structure, and on PC1 (Fig. 1). This initial lower-dimensional visualization of imaging data informed of clustering of the groups in our multivariate brain imaging dataset.

### Supervised learning

#### CTN versus HC

**Figure 2:**
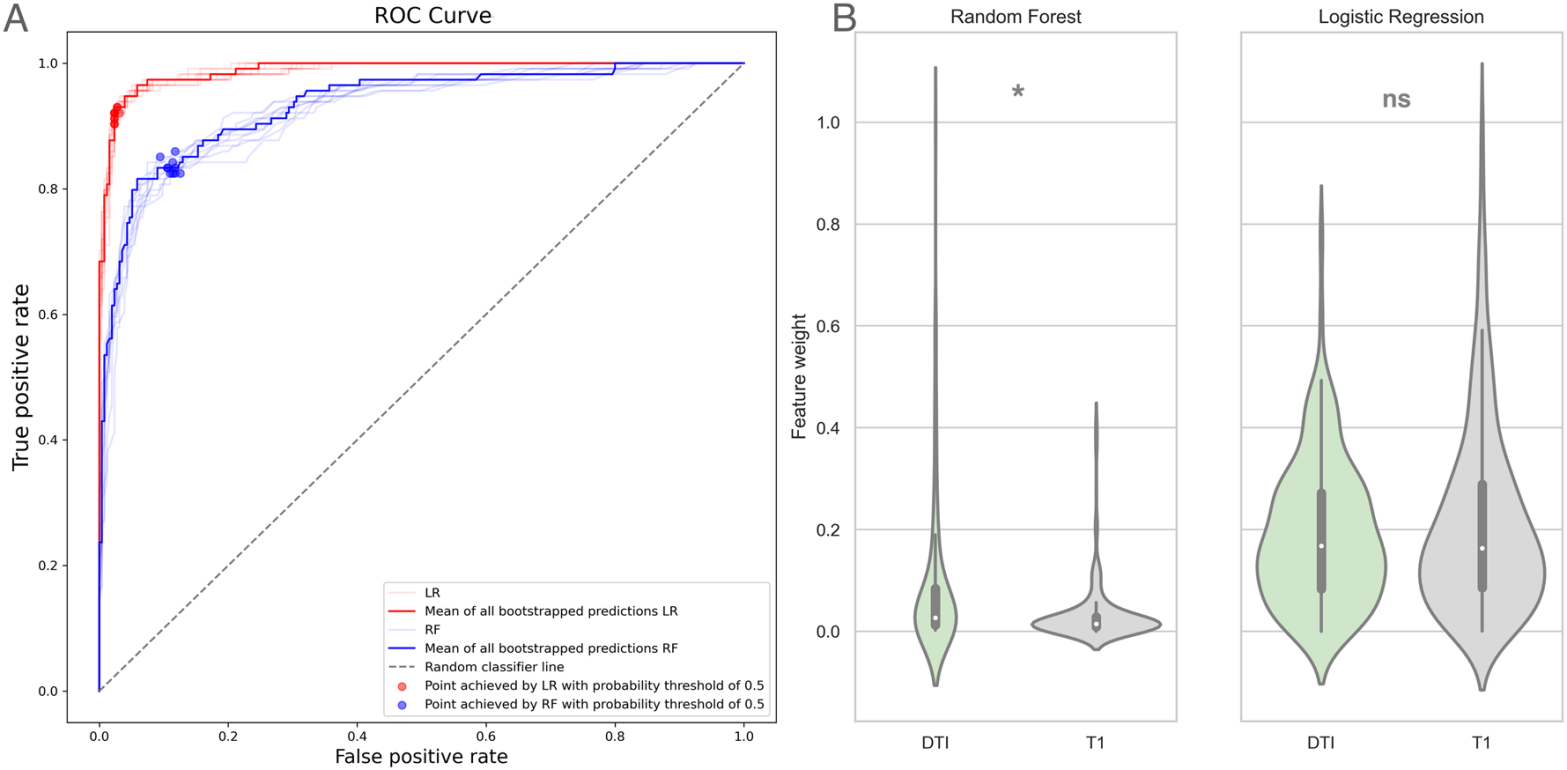
CTN versus HC classification model performance (**A**) and analysis of variance of feature weights (**B**). The areas under the ROC curves are 0.93 and 0.99 for the RF and LR, respectively (p < 0.05). The RF model prioritized DTI measures, whereas LR was agnostic regarding imaging modality. *CTN* classical trigeminal neuralgia, *HC* healthy control, *ROC* receiver operator curve, *LR* logistic regression (red ROC), *RF* random forest (blue ROC), *DTI* diffusion tensor imaging.

Random forest (RF) and bagged logistic regression (LR) performances were assessed using areas under the receiver operator curve (AUC). CTN versus HC classifier models achieved prediction accuracies of 95% for LR (CTN recall 0.97, HC recall 0.92) and 86% for RF (CTN recall 0.87, HC recall 0.84) (Fig. 2A). Analysis of variance of the brain imaging features revealed that RF prioritized diffusion imaging metrics (FA, RD, AD) for this classification task, ranking these features significantly higher compared to the T1 measures (cortical thickness, surface area, and gray matter volume) (*p* < 0.05). In contrast, there was no significant difference in the weights of T1 and DTI measures for the LR model of CTN versus HC classification (Fig. 2B).

**Figure 3:**
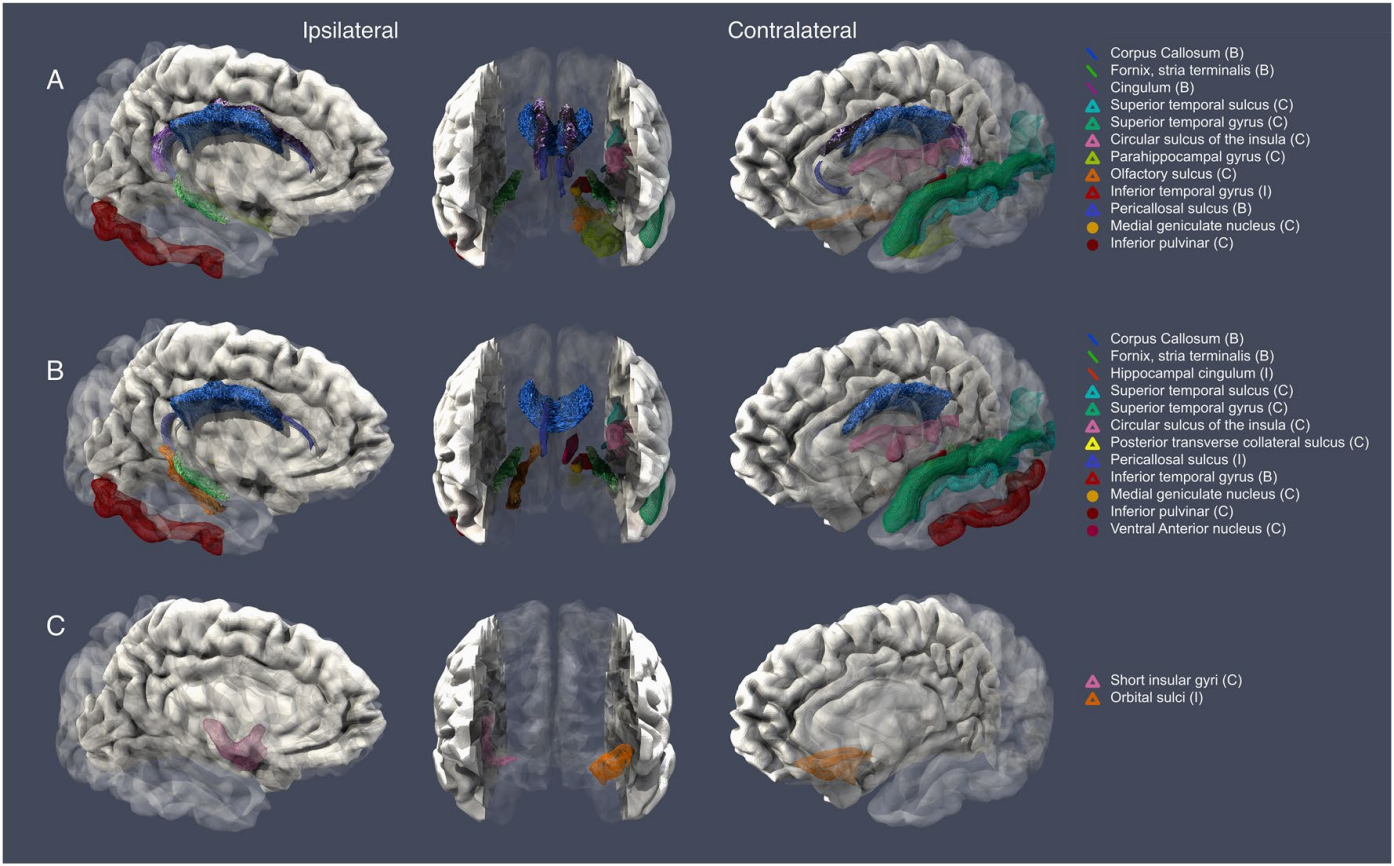
Visualization of the important MR imaging classification predictors identified by AI models, arranged according to the classification task and side of pain (ipsilateral versus contralateral to pain side). (**A**) CTN versus HC models, (**B**) TNP versus HC models, (**C**) CTN versus TNP models. Middle column depicts coronal views. Vertices correspond to the cortical thickness and surface area metrics, lines correspond to the diffusivity metrics (FA, RD, AD), and circles correspond to the thalamic nuclei volume metrics. A total of 12 neuroanatomical predictors were visualized for both LR and RF CTN versus HC models, of which 4 were bilateral, 7 contralateral and one ipsilateral. Also, 12 predictors were visualized for both LR and RF TNP versus HC models, of which 3 are bilateral, 7 were contralateral and 2 ipsilateral. Only two predictors are identified for CTN versus TNP, one contralateral and one ipsilateral. All displayed neuroanatomical predictors showed significant differences between compared groups (CTN versus HC, TNP versus HC and CTN versus TNP respectively). *HC* healthy control, *CTN* classical trigeminal neuralgia, *TNP* trigeminal neuropathic pain, *HC* healthy control. *B* Bilateral, *I* Ipsilateral to pain side, *C* Contralateral to pain side. Rendered in Paraview.

The top predictors of CTN versus HC differentiation identified with supervised learning models included gray matter thickness at the temporal area (bilaterally), contralateral insula, olfactory cortex and parahippocampal gyrus, white matter diffusivity at the fornix, corpus callosum and cingulum, and gray matter volume reductions at the contralateral inferior pulvinar and medial geniculate nuclei (see Fig. 3). Univariate statistics demonstrated significant differences between TNP and HC for 42 out of the 50 top predictors of LR and all of the top 50 features derived by RF model (*p*-corrected < 0.05) (Supplemental Table 3).

#### TNP versus HC

**Figure 4:**
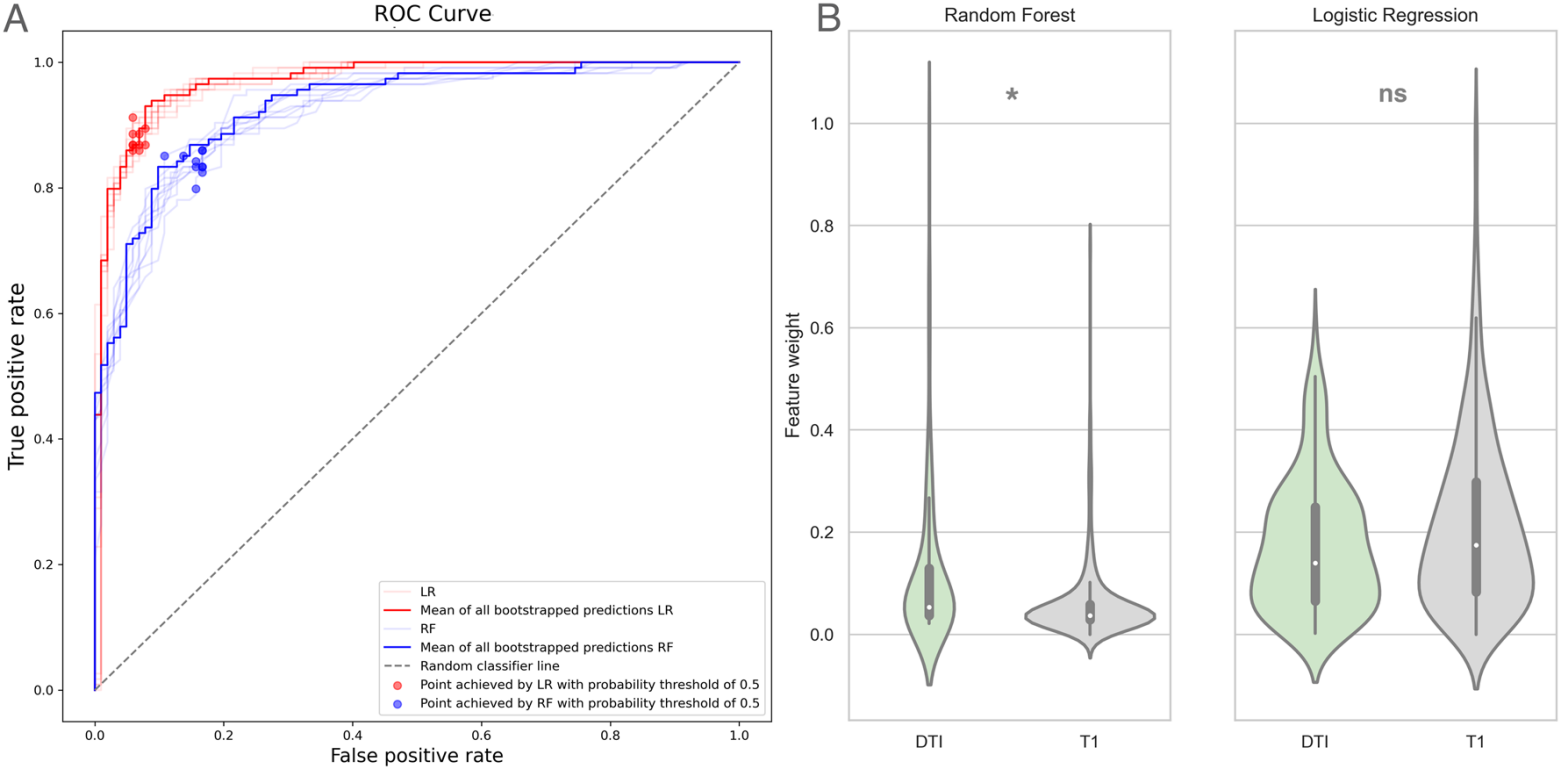
TNP versus HC classification models performance (**A**) and analysis of variance of feature weights (**B**). The areas under the ROC curves are 0.92 and 0.97 for the RF and LR, respectively (*p* < 0.05). The RF model prioritized DTI measures, whereas LR was agnostic regarding imaging modality. *TNP* trigeminal neuropathic pain, *HC* healthy control, *ROC* receiver operator curve, *LR* logistic regression (red ROC), *RF* random forest (blue ROC), *DTI* Diffusion Tensor Imaging.

Classification of TNP and HCs demonstrated 84% accuracy for the RF (TNP recall 0.85, HC recall 0.84) and 91% accuracy for the LR (TNP recall 0.93, HC recall 0.88) (Fig. 4A). The accuracy was slightly lower than the performance of the CTN versus HC classification models (Fig. 4A). Analysis of variance of the features lists showed a similar trend as the CTN versus HC models for feature weights: RF prioritized DTI measures, whereas LR demonstrated no preference with regards to imaging modality-specific metrics (Fig. 4B). Our model identified several gray and white matter metrics as predictive for distinguishing TNP from HC, including corpus callosum, fornix and hippocampal cingulum diffusivity measures, contralateral insula and temporal lobe area gray matter (superior and inferior temporal gyri) changes bilaterally, and medial geniculate, ventral anterior and inferior pulvinar nuclei gray matter volume. Regional grey matter volume, thickness and surface area was reduced in TNP comparing to HCs (Fig. 3). Univariate statistics of top 50 features revealed 39 statistically significant LR predictors derived by LR and 48 statistically significant RF predictors (Supplemental Table 4).

#### CTN versus TNP

**Figure 5:**
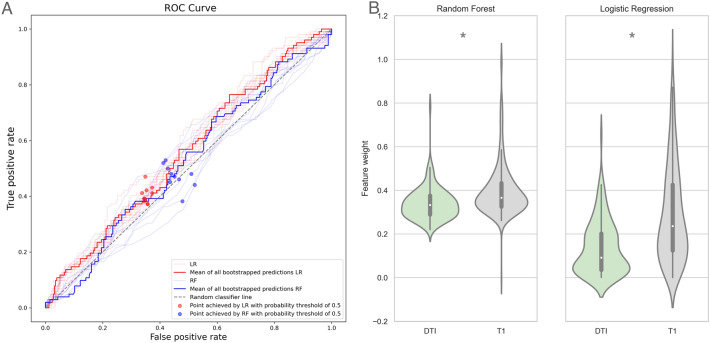
CTN versus TNP classification models performance (**A**) and analysis of variance of feature weights (**B**). The areas under the ROC curves were 0.51 and 0.55 for the RF and LR, respectively (*p* < 0.05). Both RF and LR prioritized T1 measures over DTI. *LR* logistic regression (red ROC), *RF* random forest (blue ROC), *DTI* diffusion tensor imaging.

RF and LR classifiers of CTN and TNP showed approximately 51% accuracy with AUC equal to 0.51 (RF CTN recall 0.54, TNP recall 0.47) and 0.55 (LR CTN recall 0.65, TNP recall 0.41), respectively (Fig. 5A). However, univariate statistics found significant differences between CTN and TNP (*p*-corrected < 0.05) at two cortical measures: ipsilateral insular thickness and contralateral orbital sulcus thickness (Fig. 3 and Supplemental Table 5). These structures were significantly reduced in TNP group comparing to CTN. Both RF and LR CTN versus TNP models ranked T1 measures significantly higher than DTI measures for the CTN versus TNP classification task (*p* < 0.05) (Fig. 5B).

## Discussion

In this study, we focused on the use of multimodal brain imaging data alone to explore whether AI tools can distinguish chronic neuropathic facial pain subtypes from healthy control data. We found that AI tools with brain imaging data distinguished facial pain (CTN, TNP) from healthy controls with very high accuracy (up to 95% for CTN versus HC and up to 91% for TNP versus HC). Univariate statistics confirmed the directionality of changes in neuroanatomical substrates identified in both models (CTN versus HC and TNP versus HC) between compared groups (*p*-corrected < 0.01). Thus, using multiple AI models, we were able to identify neuroanatomical features that distinguish CTN from HC and TNP from HC. Importantly, while our models could not sufficiently discriminate between CTN and TNP, the models uncovered structural differences between CTN and TNP in the ipsilateral insula and contralateral orbital cortex, with subsequent univariate statistical analysis confirming the significant differences in these structures between the CTN and TNP groups (*p*-corrected < 0.05). Using a large dataset with heterogenous brain imaging data, we demonstrate that AI models can capture structural imaging-based facial pain signatures. The use of such a large-scale, dataset—to our knowledge the largest in chronic pain literature—permits precise and generalizable AI-driven investigation of chronic facial pain signatures^20^. Our findings open the pathway for a greater level of objectivity in the assessment of chronic neuropathic facial pain conditions.

### AI for MR data analysis

AI can significantly improve the investigation of hidden factors in clinical data to obtain actionable gap-based information about patients and both prevent and rapidly detect disease^32^. This is important, especially for medical imaging, as AI is able to analyze and interpret large quantities of data without the subjective input of human factors^33^. Supervised learning offers highly focused and efficient analysis and can also highlight hidden predictive features of classified groups. In addition, AI can provide complementary insight when combined with conventional statistics—this has been demonstrated in the current study as well as in previous reports, summarized by Rajula et al.^34^.

An important requirement for AI models built with MR imaging data is their interpretability. RF and Bagged LR are ensemble methods which consist of multiple decision trees and logistic regressions, respectively. These models offer a high level of interpretability, necessary for effectively studying medical imaging data^19,22,27,35^. In addition, RF selects the best feature during training for splitting each node, therefore these classifiers work well with complex multivariate data^36^. Model performance and generalizability also depends on multiple factors, including sample size, heterogeneity of data, and the implementation of cross-validation procedures^37–39^. While larger training sample sizes increase the generalizability of study findings, it has been reported that studies with n > 150 actually suffer from lower model performance due to data heterogeneity^40^. Prior studies in chronic neuropathic facial pain used AI techniques such as support vector machines and Gaussian process classification with diffusion imaging to identify markers distinguishing CTN from HC. These studies achieved accuracies of 88% and 85% in distinguishing TN and HC subjects on relatively small datasets (46 and 72 subjects respectively)^24^. Compared to these attempts, we used different AI approaches and further refined our models to utilize multimodal (T1 and DTI) imaging data (from 371 CTN and TNP subjects and 108 healthy controls). This results in higher generalizability, and, more importantly, an increase in model performance (up to 95%).

The addition of clinical features (such as characteristics of pain presentation) to our models may further uncover the unique clinical attributes of pain syndromes. However, mixing the multimodal MR data with subjective categorical clinical variables would require the introduction of additional feature transformation approaches, potentially lowering the interpretability of the models, introducing sampling and cognitive biases to the analysis, resulting in model overfitting^41^. Therefore, our study focused on the objective multimodal MR data only as the model input.

### AI-driven brain imaging biomarkers of chronic neuropathic facial pain

Prior literature on gray and white matter alterations has revealed regional brain abnormalities in neuropathic facial pain. Reduction of overall thalamic volume and pulvinar volume has been reported previously for CTN subjects^42^. Chronic and acute pain-related blood flow changes were demonstrated on facial pain patients with thalamic deep brain stimulation and significant differences in anterior insula and pulvinar regional blood flow between pain-free and non-stimulated pain patients has been reported^43^. In addition to group-level regional differences, grey matter volume decreases in the parahippocampal gyrus and temporal lobe have been correlated with the duration of TN^44^. Abnormalities in CNS white matter, in areas such as corpus callosum, cingulum and fornix have been previously reported, and are consistent with our observation (Fig. 3)^45–47^. While previous studies have used univariate statistical approaches to focus on specific regions of interest, our current unrestricted AI methodology has identified similar white matter regions of interest. This provides an important corroboration of the neuroanatomical regions using different methods of analysis.

Furthermore, the interpretability of the AI algorithms allowed us to both identify the previously described CNS structures associated with trigeminal pain, and highlight additional regions whose role in pain and sensory modulation should be further investigated (e.g., medial geniculate nucleus, posterior transverse collateral sulcus). It is plausible, for instance, that medial geniculate nucleus abnormalities are consistent with previous reports of patients with TN having an increased risk of developing auditory system disorders such as tinnitus^48^, and the concept that chronic pain may share some elements of pathogenesis with thalamocortical dysrhythmia^49,50^. A previous AI study also objectively identified occipital gray matter as predictive for surgical treatment response in TN patients^28^. Significant differences observed in pulvinar volume might also suggest that visual circuitry could be involved in chronic pain^42^. That supervised learning AI methodology is able to pinpoint these possible associations with structural features could be the subject of more focused research in both pathogenetic mechanisms and/or therapeutic interventions.

## Orbitofrontal cortex and insula as key structures that differentiate between CTN and TNP

CTN and TNP classification models identified two important grey matter regions which demonstrated statistically significant differences between compared groups: ipsilateral insular gyri and contralateral orbitofrontal cortex. In the context of a comparison of CTN and TNP, these findings might suggest the link between severity, treatment outcomes and pain inhibition differences between these two syndromes. Insular abnormalities in chronic pain syndromes have been linked to the chronicity of pain^51,52^. Reward and pleasure-induced pain inhibition were reported to be modulated by orbitofrontal cortex areas^53–55^. De Souza et al. reported significant grey matter reduction in the insula and orbitofrontal cortex in classical and idiopathic TN patients^56,57^. Our results highlight the value of objective AI-based imaging analyses for the identification of group-level abnormalities, which may not be identifiable using univariate methods. For instance, Obermann et al. did not find morphological features differentiating constant and paroxysmal trigeminal pain subjects^44^.

While prior studies identified gray and white matter changes in TN using conventional statistical analysis methods, our study adds supervised learning AI algorithms as a modern way to investigate pain. We propose that these two sites (insula, OFC) should be studied further in the context of constant trigeminal pain. Given that patients with CTN and TNP have significant variability in pain relief after surgery, these structures may serve as potential predictors of surgical outcomes^11,58,59^.

### Limitations and future directions

Our work focuses on brain imaging as the sole data source that informs the AI algorithms; however, we note that each brain imaging dataset is in fact labelled as either CTN or TNP, which are labels that arise from a clinical diagnosis. This dichotomy is an inherent limitation of supervised learning approaches in pain. As this is the early stage of applying AI in pain, we expect that new methods will lead to more complex ways of classifying facial pain syndromes and, consequently, more complex classification models.

The poor distinction between CTN versus TNP classification (~ 51%) can be considered as a limitation of this study. The clinical diagnosis of CTN and TNP is chiefly based on the clinical expression of pain. We acknowledge that there is a Venn diagram between these two entities, and it is possible that the clinical label is not perfectly accurate. This might contribute to the limited AI-based discrimination of these entities. Furthermore, atypical forms of trigeminal pain (constant pain, deafferentation pain and postherpetic neuralgia) consist in pooled data, of what may constitute several pain conditions that are different from each other but have a similar expression of pain. Future studies can focus on larger TNP and greater distinction among these conditions, to investigate whether there are any unique structural patterns which can be identified by more complex AI tools.

This retrospective study may provide insights into how structural changes in T1 and DTI imaging are associated with specific diagnoses of chronic pain and potentially identify a novel approach to classifying trigeminal pain syndromes. As our study focuses on the cortical and subcortical gray and white matter metrics, however, it does not include measurements from the trigeminal nerve, either along the cisternal segment, root entry zone or the pons. Prior reports of trigeminal nerve level abnormalities in TN describe the role of these regions in the prediction of the type of pain or the successful surgical outcome^60,61^. Possible future addition of the trigeminal nerve diffusivity metrics to the model can further improve the model performance.

Subjects with TN secondary to multiple sclerosis (MS-TN group) were excluded from analysis due to having predominantly MS-specific MR signatures. Classification of the CTN and MS-TN groups would reveal MS/non-MS comparison and therefore highlight MS-specific demyelination-related features. Since our primary focus is the trigeminal pain-specific imaging features, the analysis of the MS-TN group compared to the pain-free MS subjects would be more appropriate for future studies.

## Conclusion

This study demonstrated the ability of AI to serve not only as a classification tool, but also as the brain imaging data analysis framework which can uncover structural insights into chronic pain. The use of AI in combination with multimodal MR imaging data may permit novel, objective data-driven patient management.

## Methods

### Study approval

This retrospective study was approved by the University Health Network (UHN) Research Ethics Board (Protocol number: 18-5780). As patient data included in the study was collected retrospectively, additional informed consent was not required according to the University Health Network Research Ethics Board policy. Healthy control participants were recruited from the community and provided written informed consent before the study. All experiments were performed in accordance with relevant guidelines and regulations.

### Data acquisition and selection

914 adult patients diagnosed with neuropathic facial pain conditions at the Toronto Western Hospital, locally followed up between 2005 and 2018, were identified as potential study subjects. All patients included in this study were diagnosed by a neurosurgeon (MH) based on the International Classification of Headache Disorders, 3rd edition (ICHD-3) and Burchiel Classification^9,62^. Of these, we initially selected 586 subjects with pre-surgical 3T T1- and diffusion-weighted imaging data as well as present pain symptoms at a time of the MR imaging acquisition. We also identified data from healthy individuals recruited locally (*n* = 50) and the publicly available Cambridge Centre for Ageing Neuroscience dataset (*n* = 58)^25,63^. An equivalence test (TOST) and t-SNE visualization were used to assess the similarity of data extracted from local cohort and CamCAN^31,64^. Exclusion criteria for the healthy control (HC) and patient data included: a history of any neurological disease, clinically evident sensory deficit, and MR data artifacts (e.g., sliced image, major processing errors resulting in a failure of the pipeline) (Supplementary Fig. 1). MS-TN subjects (n = 64) were also excluded from the further analysis given their clear DTI-based distinction from the non-MS population. After exclusion, including quality assurance of the processed T1 and DWI imaging data and verification of clinical information, data from 371 adults with neuropathic facial pain and 108 HC was used in this study.

### MRI acquisition

All patients and healthy controls included in this study underwent similar clinical imaging protocols, detailed in Table 2.

**Table 2:** Parameters of the MR imaging datasets.

Dataset	Scanner	Population	Parameters of imaging data
Local UHN dataset	3 T GE Signa HDx	TN, Chronic facial pain (n = 371)	T1: voxel size = 1 mm, matrix = 256 × 256, flip angle = 20°, FOV = 24 cm) DWI: 60 or 30 diffusion gradient directions, 1 B0, b = 1000 s/mm2, voxel size = 3 mm, matrix = 256 × 256, flip angle = 90°
Recruited healthy control	3 T GE Signa HDx	Healthy population (n = 62)	T1: voxel size = 1 mm, matrix = 256 × 256, flip angle = 20°, FOV = 24 cm DWI: 60 or 30 diffusion gradient directions, 1 B0, b = 1000 s/mm2, voxel size = 3 mm, matrix = 256 × 256, flip angle = 90°
Cam-CAN	3 T Siemens TIM Trio	Healthy population (n = 46)	T1: voxel size = 1 mm, matrix = 256 × 240, flip angle = 9°, FOV = 25.6 cm DWI: 30 diffusion gradient directions, B0, b = 1000/2000s/mm2 (b1000 shell was used). voxel size = 2 mm

*Cam-CAN* Cambridge Centre for Ageing and Neuroscience, *DWI* diffusion weighted imaging, *FOV* field of view, *TN* trigeminal neuralgia.

### MRI preprocessing

T1-weighted MR images were converted to ‘nifti’ format and processed in FreeSurfer (version 7.0; https://surfer.nmr.mgh.harvard.edu/). Cortical thickness metrics were extracted from each subject with its standard image processing and segmentation pipeline (“recon-all” function)^65,66^. Outputs of the pipeline were used to extract regional grey matter measurements (gray matter thickness, surface area, volume). Regional cortical thickness and surface area metrics for each study subject were exported using Destrieux atlas segmentation^67^. We segmented individual thalamic nuclei using “segmentThalamicNuclei” and extracted gray matter volume for each nucleus^68^. All gray matter measurements were normalized by estimated total intracranial volume for each subject.

**Figure 6:**
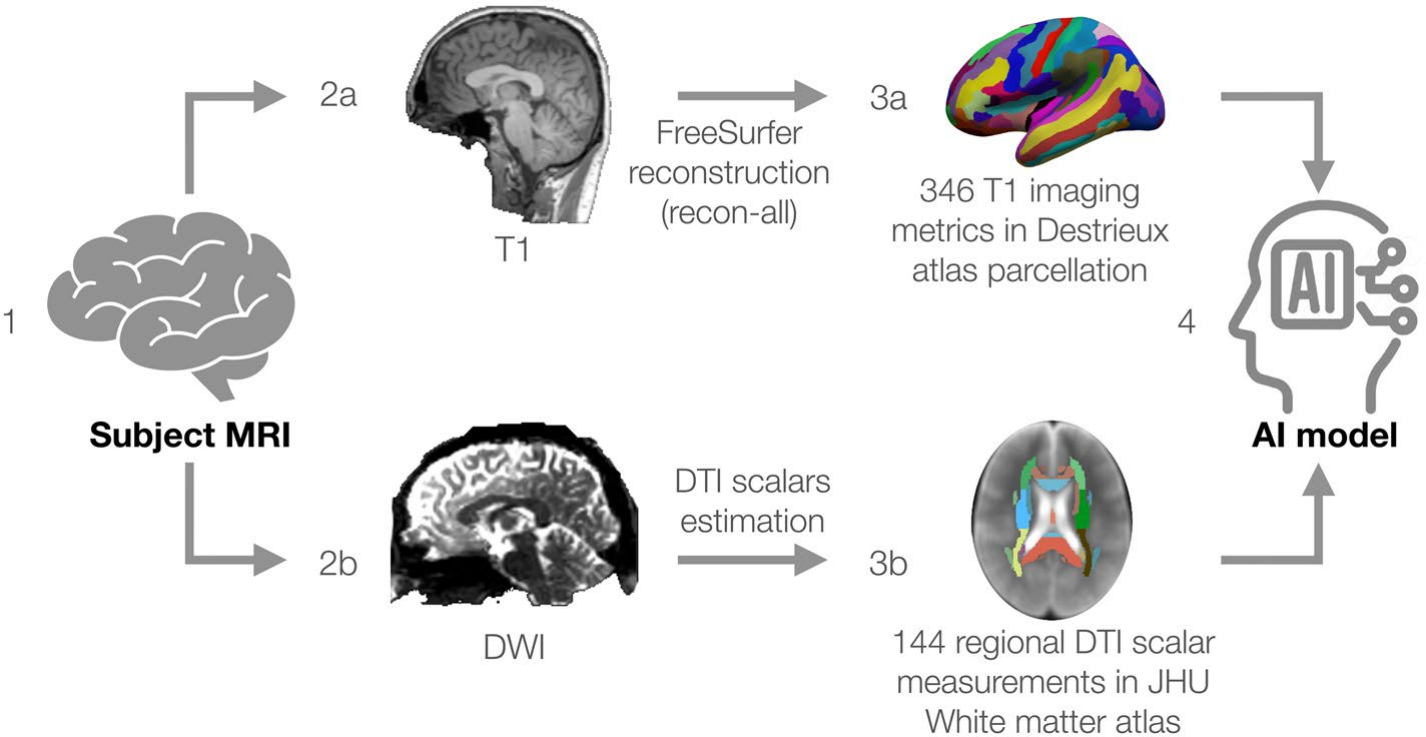
MR imaging data processing pipeline. (1) Converting the image into ‘nifti’ format; (2a) Reconstruction of T1 imaging data in FreeSurfer. (2b) DTI scalars estimation. (3a) Segmentation of T1 imaging data using Destrieux atlas. (3b) Extraction of DTI measures using JHU White Matter Atlas. (4) Importing the T1 and DTI data to the artificial intelligence (AI) model. *DTI* diffusion tensor imaging, *JHU* Johns Hopkins University.

Diffusion-weighted images underwent eddy current, motion correction and skull-stripping in FSL (https://fsl.fmrib.ox.ac.uk/fsl/fslwiki), followed by diffusion tensor estimation and derivation of three white matter diffusivity scalars; fractional anisotropy (FA), radial diffusivity (RD), and axial diffusivity (AD) using 3D Slicer (SlicerDMRI, https://www.slicer.org/). These DTI scalar volumes were aligned with the Montreal Neurological Institute (MNI) standard template space by nonlinear registration using Advanced Normalization Tools (ANTs; http://stnava.github.io/ANTs/)^69^. The Johns Hopkins University (JHU) White Matter Atlas was then used to extract regional DTI metrics for each scalar volume^70^. A summary of all imaging data processing is shown in Fig. 6.

All brain imaging features in the dataset were first standardized to unit variance and labelled with respect to pain laterality (ipsilateral or contralateral) to facilitate subsequent supervised and unsupervised ML analyses. This improves the generalizability and interpretability of constructed models^71^. If the subject is a healthy control, the ipsilateral hemisphere was randomly chosen from either the left or right hemispheres. This has been done to ensure that HC data follow the same transformation as the subjects in the pain dataset. All 490 features were used to perform unsupervised and supervised ML tasks.

### Artificial intelligence models

#### Unsupervised learning

We used dimensionality reduction algorithms to overcome the limitations of conventional voxel-wise statistics-based methods and to identify brain imaging data patterns that correspond to different chronic facial pain classes within our subject cohort. Specifically, we used principal component analysis (PCA) and t-distributed stochastic neighbour embedding (t-SNE)^64,72^, both of which allow a large number of redundant metrics to be condensed into relatively few new variables, which carry the data-representative information and can be easily visualized^73^.

A two-dimensional t-SNE was used to visualize the dataset. PCA was used to reduce the dimensionality of the dataset. The most important features in the PCA were defined as those with the highest absolute values for the PCA loadings for a particular component (PC1). PC1 of facial pain and healthy control subjects were compared using Student’s independent *t*-test under the null hypothesis of no difference between compared distributions.

#### Supervised learning

We used two supervised learning methods—balanced Random Forest (RF) and bagged Logistic Regression (LR). The RF classifier model was selected given our multivariate data and the RF’s inherent ability to perform feature selection^36^. The metrics used to evaluate the models were: accuracy, recall, precision, and the receiver operating characteristic (ROC) curve. Default hyperparameters were used (Supplemental Table 1). The most important features were defined as the top 10 predictors that resulted in the highest mean impurity decrease in the RF classification.

We used bagged LR as a second classification algorithm. To mitigate class imbalance, a bagging classifier consisting of 10 regressions was trained, with each LR trained on a subset of the data that was randomly under-sampled from the majority class. Regressions were implemented in *scikit-learn* (Python), while the balanced bagging classifier was implemented in *imbalanced-learn*^74,75^. Usage of the bagged LR and RF with the tabulated data extracted from MR imaging allows for the interpretability of the models, which is a strong advantage compared to “black box” deep learning models and raw imaging data^76,77^.

We used leave-one-out cross-validation for all tasks. On each iteration, before training, we normalized all features in the training dataset to attempt to fit a standard normal distribution. To ensure that features are scaled to the same magnitude, the testing example was transformed using the mean and standard deviations for each feature found in the training set. This process was repeated 10 times for each classifier. Mean cross-validated prediction from all 10 training iterations was used to generate an overall ROC curve for each classifier and the mean accuracy had been reported.

Feature weights for the models were stratified by the imaging modality and compared using one-way analysis of variance (ANOVA) to identify whether the model prioritizes one MR modality over the other.

#### Post-hoc univariate statistics

Following the supervised learning tasks, we performed a post-hoc univariate statistical analysis of the top 50 predictors identified by each classifier, to identify the directionalities of regional gray and white matter alterations contributing to each model prediction. The cut-off was arbitrary and defined based on the feature weights decay of classifier models (Supplemental Fig. 5). Student’s independent *t*-tests were used to determine the *p*-value under the null hypothesis of no difference between groups (CTN versus TNP, CTN versus HC, and TNP versus HC), as well as the confidence interval for *α* = *0.05*. To correct *p*-values for the use of multiple tests, the Holm-Sidak method was used to maintain a false discovery rate of 0.05 (the confidence intervals remained uncorrected). Statistically significant regions from 10 predictors of each classifier were used for visualization purposes.

## Supplementary Information

Supplementary Information.

## Data Availability

MR data from trigeminal pain subjects and code will be shared upon reasonable request. The CamCAN imaging data is publicly available (https://camcan-archive.mrc-cbu.cam.ac.uk/dataaccess/index.php).

## Abbreviations

AD: Axial diffusivity
AI: Artificial intelligence
CamCAN: Cambridge Centre for Ageing and Neuroscience
CNS: Central nervous system
CTN: Classical trigeminal neuralgia
DTI: Diffusion tensor imaging
DWI: Diffusion-weighted imaging
FA: Fractional anisotropy
ICHD-3: The International Classification of Headache Disorders 3rd edition
JHU: Johns Hopkins University
LR: Logistic regression
ML: Machine learning
MRI: Magnetic resonance imaging
PCA: Principal component analysis
RD: Radial diffusivity
RF: Random forest
ROC: Receiver operator curve
SD: Standard deviation
TN: Trigeminal neuralgia
TNP: Trigeminal neuropathic pain
t-SNE: T-distributed Stochastic Neighbor Embedding
