# Supplementary material for: Brain imaging signatures of neuropathic facial pain derived by artificial intelligence
Source: Sci Rep. 2023 Jul 3;13:10699. doi: 10.1038/s41598-023-37034-y (PMC10318015; doi:10.1038/s41598-023-37034-y)
Supplement: Supplementary file 1 — Supplementary Information. [file 41598_2023_37034_MOESM1_ESM.docx]

# Supplementary material

**
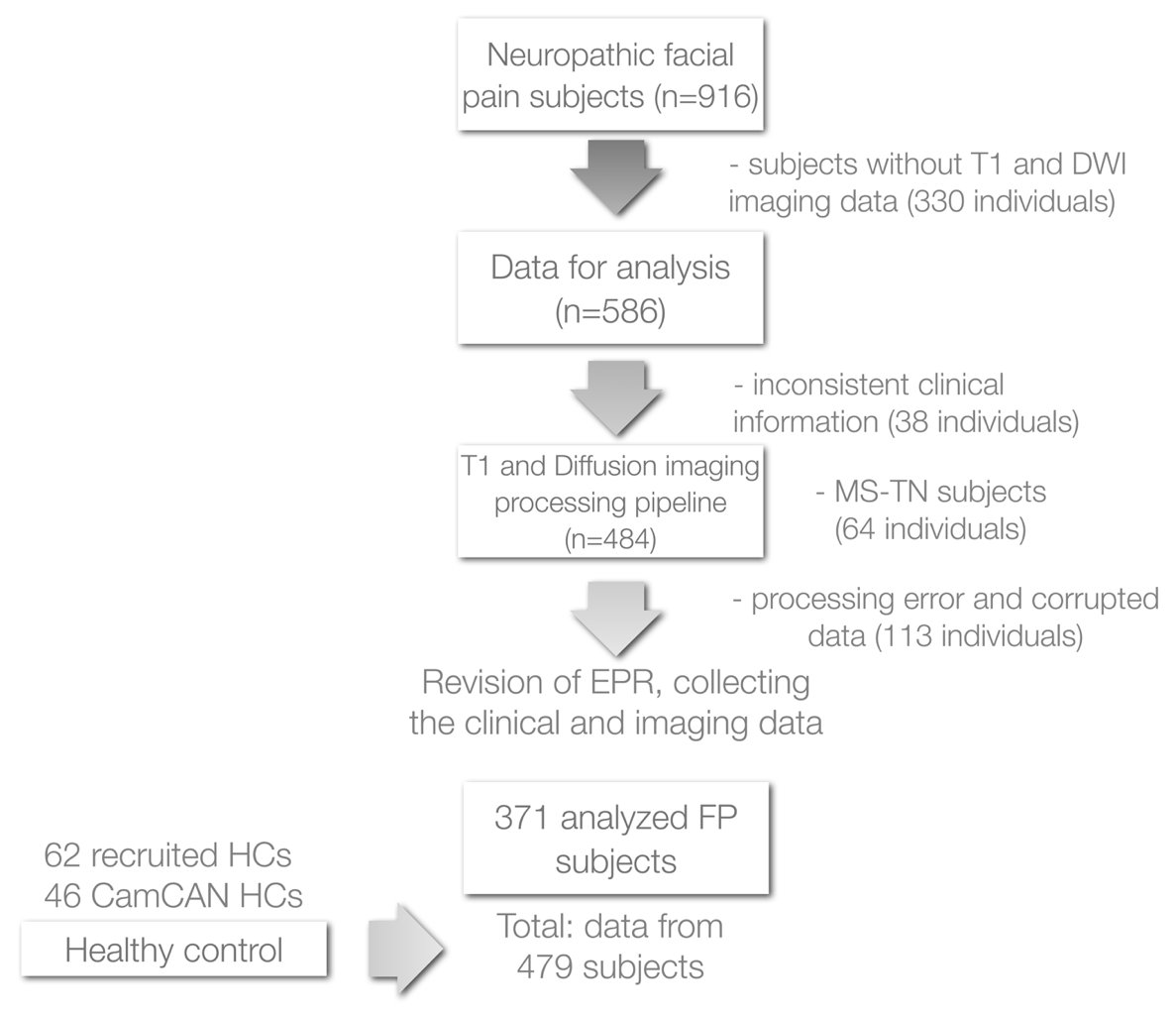
**

**Supplemental figure 1**. Flow diagram of the inclusion/exclusion of participants. CamCAN = Cambridge Centre for Ageing Neuroscience dataset, EPR – electronic patient records, FP – facial pain, HC – healthy controls, TN – trigeminal neuralgia.


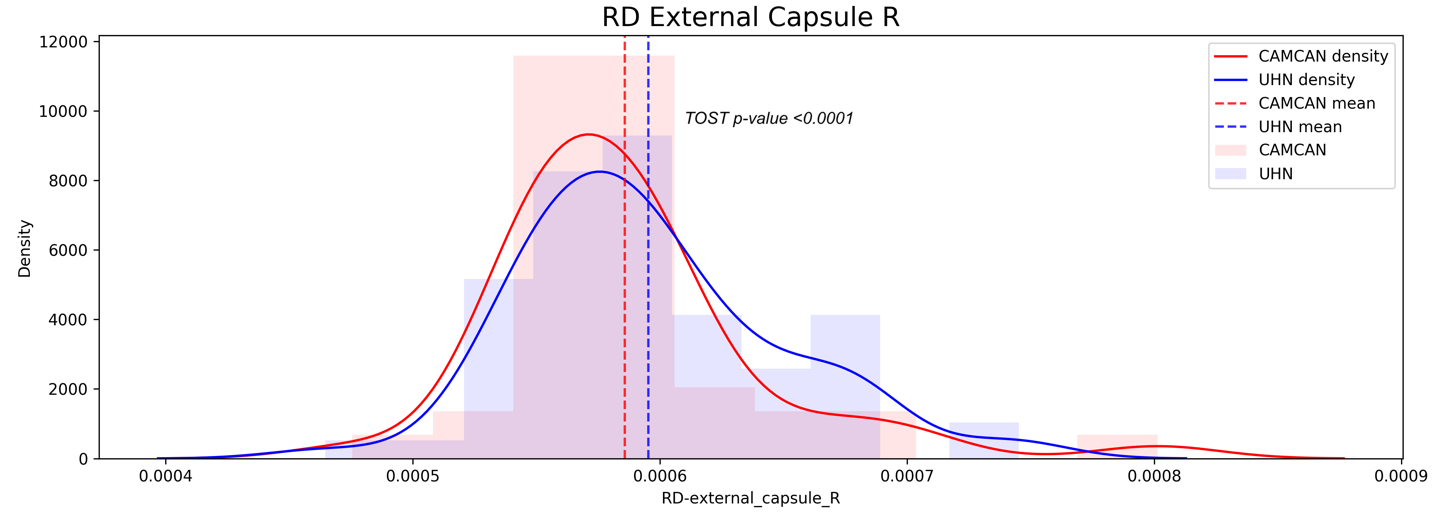


**Supplemental figure 2**. Two one-sided *t*-tests procedure shows the significant equivalence of regional intensity distribution for local (UHN) and external (CAMCAN) cohorts.


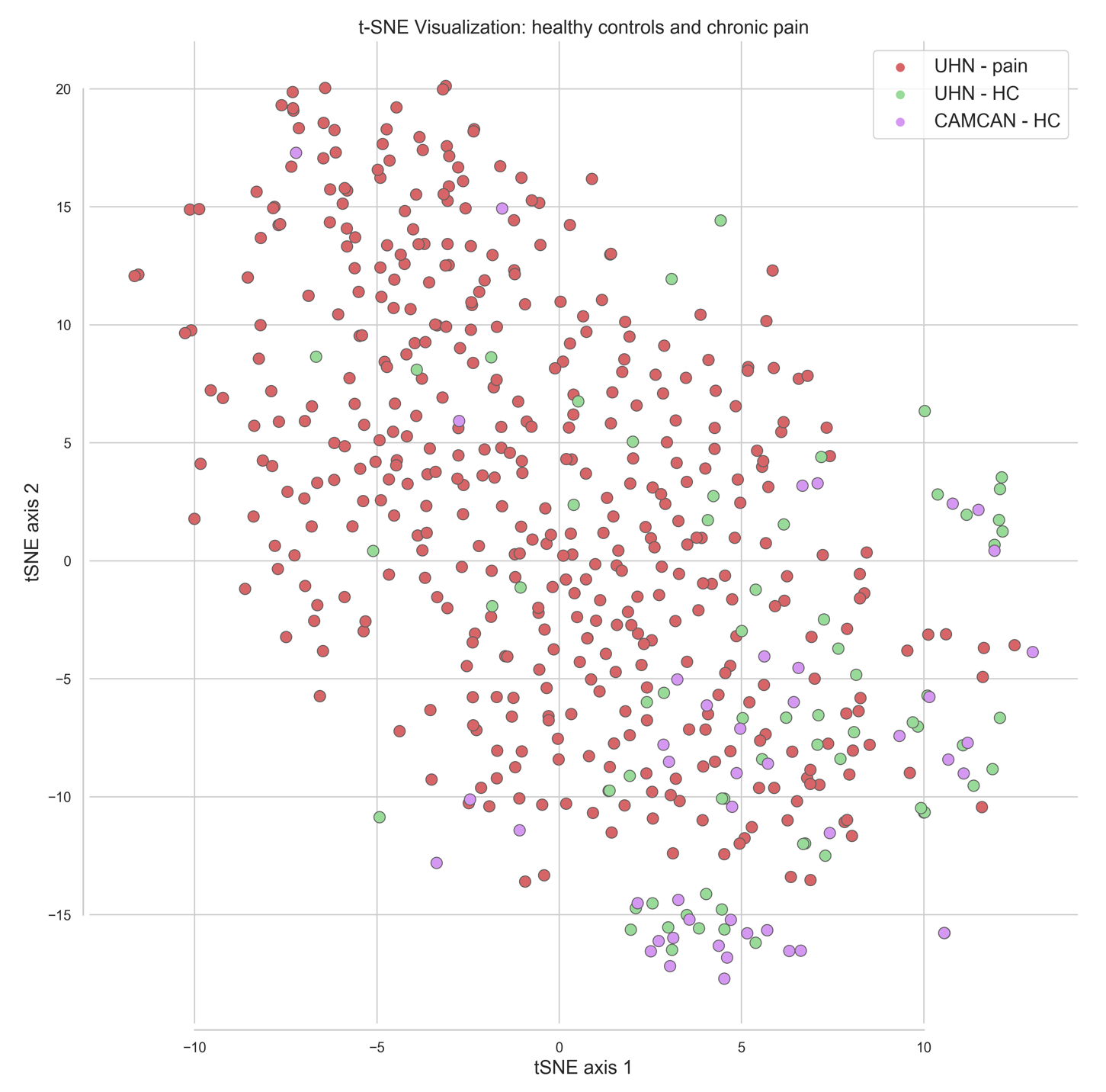


**Supplemental figure 3**. T-SNE visualization of data without the standardization for the pain side. Healthy controls (internal and external) are forming a single cluster.


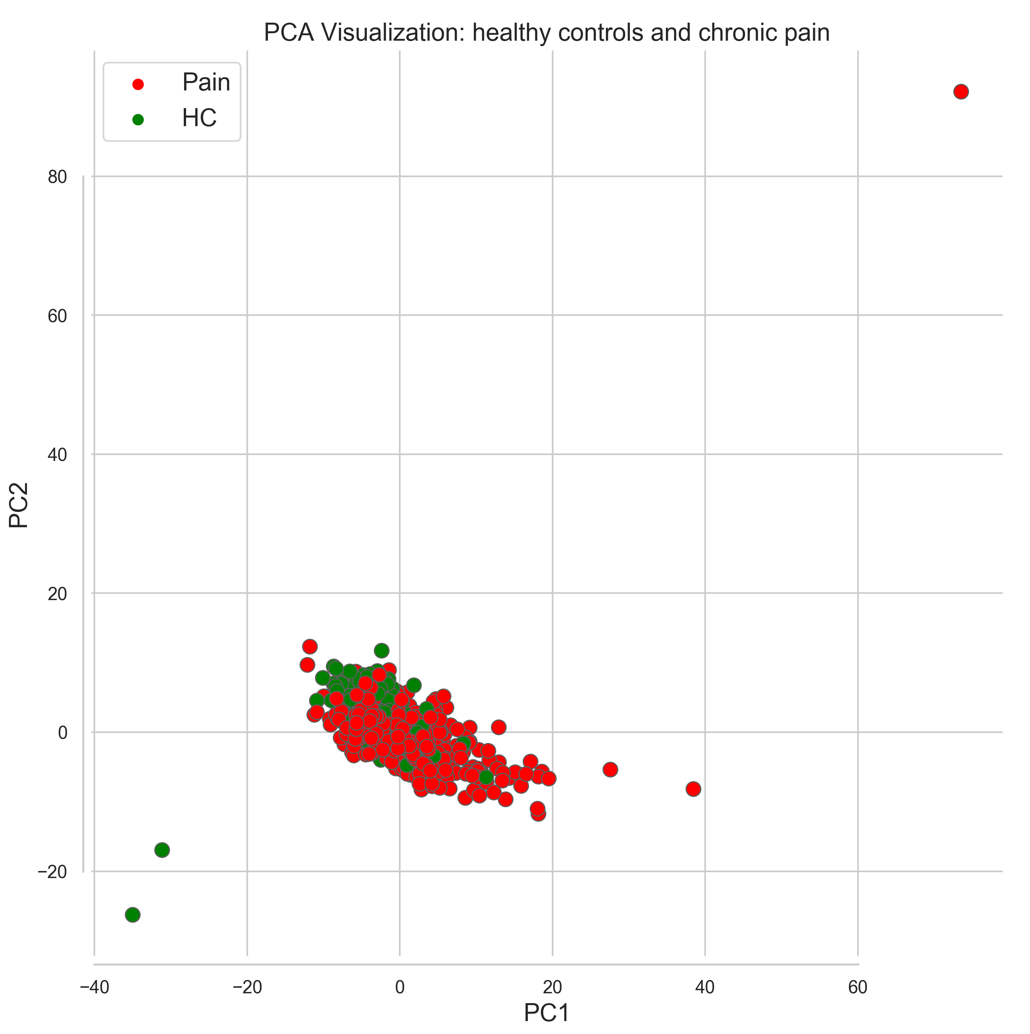


**Supplemental figure 4**. PCA visualization of the dataset. PC1 and PC2 were used for 2D visualization.


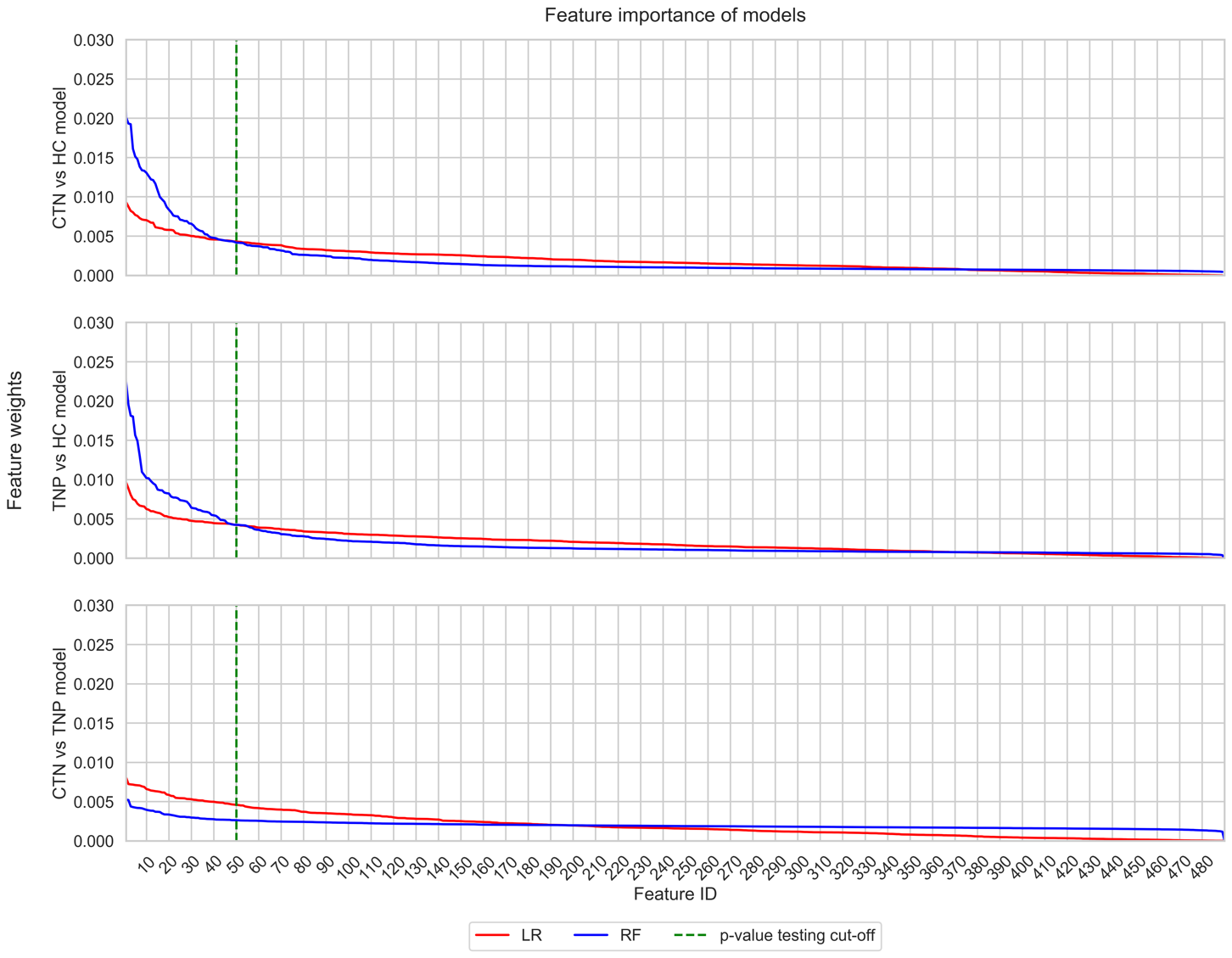


**Supplemental figure 5**. Distribution of feature weights for 3 machine learning classifiers. The green line shows the threshold of the number of features used for post-hoc statistical testing (50) based on the decay of feature weights.

| **Model** | **Parameters** |
| --- | --- |
| Random Forest | n_estimators=100, criterion='gini', max_depth=None, min_samples_split=2, min_samples_leaf=1, min_weight_fraction_leaf=0.0, max_features='auto', max_leaf_nodes=None, min_impurity_decrease=0.0, bootstrap=True, oob_score=False, sampling_strategy='auto', replacement=False, n_jobs=None, random_state=None, verbose=0, warm_start=False, class_weight=None, ccp_alpha=0.0, max_samples=None |
| Bagged Logistic Regression | penalty='l2’, dual=False, tol=0.0001, C=1.0, fit_intercept=True, intercept_scaling=1, class_weight=None, random_state=None, solver='lbfgs', max_iter=100, multi_class='auto', verbose=0, warm_start=False, n_jobs=None, l1_ratio=None, n_estimators=10 |

**Supplemental Table 1.** Hyperparameters of the models.

| **MR imaging metric group** | **Number of metrics** | **p-value of TOST** |
| --- | --- | --- |
| Cortical thickness of Destrieux atlas regions | 148 | <0.00001 |
| Cortical surface area of Destrieux atlas regions | 148 | <0.00001 |
| Thalamic nuclei volume | 50 | <0.00001 |
| DTI FA from JHU WM Atlas | 48 | <0.0001 |
| DTI AD from JHU WM Atlas | 48 | <0.0001 |
| DTI RD from JHU WM Atlas | 48 | <0.0001 |

**Supplemental Table 2.** Results of the two-one sided *t*-test procedure between the local HC subset and age/sex matched controls from CamCAN dataset.

| Metric identified as predictor | P-corrected | Model |
| --- | --- | --- |
| AD-cerebral_peduncle_contra | < 0.0001 | LR&RF |
| contra_G_oc-temp_med-Lingual_thickness | < 0.0001 | LR&RF |
| contra_G_temp_sup-Lateral_thickness | < 0.0001 | LR&RF |
| contra_S_circular_insula_ant_thickness | 0.0009 | LR&RF |
| contra_S_circular_insula_sup_thickness | < 0.0001 | LR&RF |
| contra_S_orbital_med-olfact_thickness | 0.0003 | LR&RF |
| contra_S_temporal_sup_thickness | < 0.0001 | LR&RF |
| FA-body_of_corpus_callosum | < 0.0001 | LR&RF |
| FA-cingulum_cingulate_gyrus_ipsi | < 0.0001 | LR&RF |
| FA-fornix-cres_stria_terminalis_contra | < 0.0001 | LR&RF |
| FA-fornix-cres_stria_terminalis_ipsi | < 0.0001 | LR&RF |
| ipsi_G_oc-temp_med-Lingual_thickness | < 0.0001 | LR&RF |
| ipsi_G_temp_sup-Lateral_thickness | < 0.0001 | LR&RF |
| ipsi_Lat_Fis-post_thickness | < 0.0001 | LR&RF |
| ipsi_S_calcarine_thickness | < 0.0001 | LR&RF |
| AD-cerebral_peduncle_ipsi | < 0.0001 | LR |
| CM_ipsi | 0.0003 | LR |
| contra_G_oc-temp_med-Parahip_thickness | 0.0249 | LR |
| contra_G_pariet_inf-Supramar_thickness | 0.0009 | LR |
| contra_G_precuneus_thickness | 0.0398 | LR |
| contra_G_rectus_thickness | 0.7056 | LR |
| contra_G_temporal_inf_thickness | < 0.0001 | LR |
| contra_G&S_occipital_inf_thickness | 0.0009 | LR |
| contra_S_collat_transv_post_thickness | 0.0182 | LR |
| contra_S_oc-temp_med&Lingual_thickness | 0.0102 | LR |
| contra_S_pericallosal_thickness | < 0.0001 | LR |
| FA-tapetum_contra | 0.0003 | LR |
| ipsi_G_front_inf-Orbital_area | 0.0871 | LR |
| ipsi_G_front_middle_thickness | 0.6675 | LR |
| ipsi_G_rectus_thickness | 0.2932 | LR |
| ipsi_G_temp_sup-G_T_transv_thickness | 0.0002 | LR |
| ipsi_G_temporal_inf_thickness | < 0.0001 | LR |
| ipsi_G&S_cingul-Ant_thickness | 0.3181 | LR |
| ipsi_Lat_Fis-post_area | 0.0004 | LR |
| ipsi_S_circular_insula_ant_thickness | 0.0002 | LR |
| ipsi_S_circular_insula_sup_thickness | < 0.0001 | LR |
| ipsi_S_front_inf_thickness | 0.0871 | LR |
| ipsi_S_orbital_med-olfact_thickness | 0.0004 | LR |
| ipsi_S_orbital-H_Shaped_area | 0.5865 | LR |
| ipsi_S_pericallosal_thickness | < 0.0001 | LR |
| ipsi_S_temporal_sup_thickness | < 0.0001 | LR |
| MDl_contra | 0.0001 | LR |
| MDm_contra | 0.3967 | LR |
| VPL_contra | < 0.0001 | LR |
| L-Sg_contra | < 0.0001 | LR&RF |
| MDl_ipsi | 0.0003 | LR&RF |
| MGN_contra | < 0.0001 | LR&RF |
| PuI_contra | < 0.0001 | LR&RF |
| RD-middle_cerebellar_peduncle | < 0.0001 | LR&RF |
| VA_contra | < 0.0001 | LR&RF |
| AD-pontine_crossing_tract-part_-of_-MCP | 0.0216 | RF |
| contra_G_cuneus_thickness | < 0.0001 | RF |
| FA-cingulum_cingulate_gyrus_contra | < 0.0001 | RF |
| FA-cingulum_hippo_contra | < 0.0001 | RF |
| FA-cingulum_hippo_ipsi | < 0.0001 | RF |
| FA-genu_of_corpus_callosum | < 0.0001 | RF |
| FA-middle_cerebellar_peduncle | < 0.0001 | RF |
| FA-posterior_thalamic_radiation_contra | < 0.0001 | RF |
| FA-posterior_thalamic_radiation_ipsi | < 0.0001 | RF |
| FA-sagittal_stratum_contra | < 0.0001 | RF |
| FA-sagittal_stratum_ipsi | < 0.0001 | RF |
| FA-splenium_of_corpus_callosum | < 0.0001 | RF |
| FA-superior_corona_radiata_contra | < 0.0001 | RF |
| FA-superior_corona_radiata_ipsi | < 0.0001 | RF |
| FA-superior_longitudinal_fasciculus_contra | < 0.0001 | RF |
| FA-superior_longitudinal_fasciculus_ipsi | < 0.0001 | RF |
| ipsi_S_circular_insula_sup_thickness | < 0.0001 | RF |
| ipsi_S_pericallosal_thickness | < 0.0001 | RF |
| MGN_ipsi | < 0.0001 | RF |
| RD-anterior_limb_of_internal_capsule_contra | < 0.0001 | RF |
| RD-anterior_limb_of_internal_capsule_ipsi | < 0.0001 | RF |
| RD-body_of_corpus_callosum | < 0.0001 | RF |
| RD-cerebral_peduncle_contra | < 0.0001 | RF |
| RD-cingulum_cingulate_gyrus_contra | < 0.0001 | RF |
| RD-cingulum_cingulate_gyrus_ipsi | < 0.0001 | RF |
| RD-genu_of_corpus_callosum | 0.0011 | RF |
| RD-superior_corona_radiata_contra | < 0.0001 | RF |
| RD-superior_longitudinal_fasciculus_contra | < 0.0001 | RF |
| VM_contra | < 0.0001 | RF |

**Supplemental Table 3.** Top 50 most important predictors and univariate statistics for CTN vs HC classification task. AD – axial diffusivity, FA – fractional anisotropy, HC – healthy controls, LR – logistic regression, RD – radial diffusivity, RF – Random Forest, TN – trigeminal neuralgia.

| Metric identified as predictor | P-correctied | Model |
| --- | --- | --- |
| AD-cerebral_peduncle_contra | 0.0055 | LR&RF |
| AD-cingulum_hippo_ipsi | 0.0026 | LR&RF |
| AD-pontine_crossing_tract-part_-of_-MCP | 0.0193 | RF |
| AD-tapetum_ipsi | 0.8729 | LR |
| contra_G_oc-temp_med-Lingual_thickness | < 0.0001 | LR&RF |
| contra_G_orbital_thickness | 0.043 | RF |
| contra_G_precuneus_thickness | 0.3072 | LR |
| contra_G_rectus_thickness | 0.8804 | LR |
| contra_G_temp_sup-Lateral_thickness | 0.0002 | LR |
| contra_G_temporal_inf_thickness | < 0.0001 | LR&RF |
| contra_G&S_occipital_inf_thickness | 0.0144 | LR |
| contra_Pole_temporal_thickness | < 0.0001 | LR |
| contra_S_calcarine_area | 0.2349 | LR |
| contra_S_circular_insula_sup_thickness | < 0.0001 | LR&RF |
| contra_S_collat_transv_post_thickness | 0.0027 | LR&RF |
| contra_S_front_sup_area | 0.0025 | LR |
| contra_S_orbital_med-olfact_thickness | 0.0001 | RF |
| contra_S_orbital_med-olfact_thickness | 0.0002 | LR |
| contra_S_pericallosal_thickness | 0.0007 | LR |
| contra_S_temporal_sup_thickness | < 0.0001 | LR&RF |
| FA-body_of_corpus_callosum | < 0.0001 | LR&RF |
| FA-cingulum_cingulate_gyrus_contra | < 0.0001 | LR&RF |
| FA-cingulum_cingulate_gyrus_ipsi | < 0.0001 | RF |
| FA-cingulum_hippo_contra | < 0.0001 | RF |
| FA-cingulum_hippo_ipsi | < 0.0001 | RF |
| FA-fornix-cres_stria_terminalis_contra | < 0.0001 | LR&RF |
| FA-fornix-cres_stria_terminalis_ipsi | < 0.0001 | RF |
| FA-genu_of_corpus_callosum | 0.0012 | RF |
| FA-middle_cerebellar_peduncle | 0.0008 | RF |
| FA-posterior_thalamic_radiation_contra | < 0.0001 | RF |
| FA-posterior_thalamic_radiation_ipsi | < 0.0001 | RF |
| FA-sagittal_stratum_contra | < 0.0001 | RF |
| FA-sagittal_stratum_ipsi | < 0.0001 | RF |
| FA-splenium_of_corpus_callosum | < 0.0001 | RF |
| FA-superior_corona_radiata_ipsi | 0.0002 | RF |
| FA-superior_longitudinal_fasciculus_contra | < 0.0001 | RF |
| FA-superior_longitudinal_fasciculus_ipsi | < 0.0001 | RF |
| ipsi_G_cuneus_thickness | < 0.0001 | LR&RF |
| ipsi_G_front_inf-Orbital_area | 0.1047 | LR |
| ipsi_G_oc-temp_lat-fusifor_thickness | 0.0027 | LR |
| ipsi_G_oc-temp_med-Lingual_thickness | < 0.0001 | LR&RF |
| ipsi_G_orbital_thickness | 0.1701 | RF |
| ipsi_G_precuneus_thickness | 0.1278 | LR |
| ipsi_G_rectus_thickness | 0.2349 | LR |
| ipsi_G_temp_sup-G_T_transv_thickness | 0.0044 | LR |
| ipsi_G_temp_sup-Lateral_thickness | < 0.0001 | LR |
| ipsi_G_temporal_inf_area | 0.0017 | LR&RF |
| ipsi_G_temporal_inf_thickness | < 0.0001 | LR&RF |
| ipsi_Lat_Fis-post_thickness | < 0.0001 | LR |
| ipsi_S_circular_insula_sup_thickness | < 0.0001 | LR&RF |
| ipsi_S_front_inf_thickness | 0.0007 | LR |
| ipsi_S_intrapariet&P_trans_area | 0.0134 | LR |
| ipsi_S_oc-temp_lat_area | 0.1278 | LR |
| ipsi_S_orbital_med-olfact_thickness | < 0.0001 | LR |
| ipsi_S_orbital-H_Shaped_area | 0.2344 | LR |
| ipsi_S_pericallosal_thickness | < 0.0001 | LR&RF |
| L-Sg_contra | < 0.0001 | LR |
| MDl_contra | < 0.0001 | RF |
| MDm_contra | 0.0157 | LR |
| MGN_ipsi | 0.001 | LR&RF |
| MGN_contra | < 0.0001 | LR&RF |
| PuI_ipsi | 0.2457 | RF |
| PuI_contra | < 0.0001 | LR&RF |
| PuL_contra | 0.0059 | LR |
| PuM_contra | < 0.0001 | LR |
| RD-anterior_limb_of_internal_capsule_contra | < 0.0001 | RF |
| RD-body_of_corpus_callosum | < 0.0001 | LR&RF |
| RD-cingulum_cingulate_gyrus_contra | < 0.0001 | LR&RF |
| RD-cingulum_cingulate_gyrus_ipsi | < 0.0001 | LR&RF |
| RD-genu_of_corpus_callosum | 0.0487 | RF |
| RD-posterior_thalamic_radiation_contra | 0.001 | RF |
| RD-superior_corona_radiata_contra | < 0.0001 | RF |
| RD-superior_corona_radiata_ipsi | < 0.0001 | RF |
| RD-superior_longitudinal_fasciculus_contra | < 0.0001 | RF |
| RD-tapetum_ipsi | 0.8838 | LR |
| VA_contra | < 0.0001 | LR&RF |
| VM_contra | 0.0001 | RF |

**Supplemental Table 4.** Top 50 most important predictors and univariate statistics for TNP vs HC classification task. AD – axial diffusivity, FA – fractional anisotropy, HC – healthy controls, LR – logistic regression, RD – radial diffusivity, RF – Random Forest, TNP – trigeminal neuropathic pain.

| Metric identified as predictor | p-corrected | Model |
| --- | --- | --- |
| AD-external_capsule_contra | 0.9946 | RF |
| AD-inferior_cerebellar_peduncle_contra | 0.9946 | RF |
| AD-tapetum_ipsi | 0.3933 | LR&RF |
| AV_ipsi | 0.9979 | LR |
| CM_contra | 0.8276 | RF |
| contra_G_front_inf-Orbital_thickness | 0.9946 | RF |
| contra_G_front_inf-Triangul_thickness | 0.9979 | LR&RF |
| contra_G_front_middle_thickness | 0.9873 | LR |
| contra_G_Ins_lg&S_cent_ins_area | 0.7325 | RF |
| contra_G_insular_short_area | 0.889 | LR&RF |
| contra_G_oc-temp_lat-fusifor_area | 0.9979 | LR |
| contra_G_oc-temp_med-Parahip_thickness | 0.9979 | LR |
| contra_G_pariet_inf-Angular_area | 0.4578 | LR&RF |
| contra_G_pariet_inf-Supramar_area | 0.7145 | RF |
| contra_G_pariet_inf-Supramar_thickness | 0.6856 | RF |
| contra_G_precentral_area | 0.9979 | LR&RF |
| contra_G_precentral_thickness | 0.9979 | LR&RF |
| contra_G_temporal_middle_area | 0.7589 | LR |
| contra_G&S_cingul-Mid-Ant_area | 0.8276 | RF |
| contra_G&S_cingul-Mid-Ant_thickness | 0.9946 | RF |
| contra_G&S_cingul-Mid-Post_thickness | 0.9979 | LR&RF |
| contra_Pole_temporal_thickness | 0.3002 | LR&RF |
| contra_S_cingul-Marginalis_thickness | 0.9946 | RF |
| contra_S_collat_transv_ant_area | 0.9979 | LR&RF |
| contra_S_collat_transv_post_thickness | 0.9946 | RF |
| contra_S_front_middle_thickness | 0.6822 | LR |
| contra_S_interm_prim-Jensen_thickness | 0.9946 | RF |
| contra_S_oc_middle&Lunatus_area | 0.9979 | LR |
| contra_S_oc-temp_med&Lingual_thickness | 0.3002 | LR&RF |
| contra_S_occipital_ant_area | 0.1999 | LR&RF |
| contra_S_orbital-H_Shaped_thickness | 0.0154 | LR&RF |
| contra_S_parieto_occipital_thickness | 0.4676 | LR&RF |
| contra_S_pericallosal_thickness | 0.9979 | LR |
| contra_S_precentral-sup-part_area | 0.9979 | LR&RF |
| contra_S_subparietal_thickness | 0.9355 | LR&RF |
| ipsi_G_cingul-Post-dorsal_thickness | 0.7796 | LR&RF |
| ipsi_G_cingul-Post-ventral_area | 0.3459 | LR&RF |
| ipsi_G_front_inf-Opercular_thickness | 0.3962 | LR |
| ipsi_G_front_inf-Triangul_area | 0.9391 | LR&RF |
| ipsi_G_insular_short_area | 0.9964 | LR&RF |
| ipsi_G_insular_short_thickness | 0.0062 | LR&RF |
| ipsi_G_oc-temp_lat-fusifor_area | 0.9979 | LR&RF |
| ipsi_G_oc-temp_lat-fusifor_thickness | 0.9979 | LR |
| ipsi_G_oc-temp_med-Parahip_thickness | 0.9791 | LR |
| ipsi_G_orbital_thickness | 0.9979 | LR |
| ipsi_G_pariet_inf-Angular_area | 0.9953 | LR |
| ipsi_G_pariet_inf-Angular_thickness | 0.9979 | LR |
| ipsi_G_pariet_inf-Supramar_area | 0.9946 | RF |
| ipsi_G_pariet_inf-Supramar_thickness | 0.9635 | LR&RF |
| ipsi_G_postcentral_thickness | 0.9978 | LR&RF |
| ipsi_G_precuneus_thickness | 0.9963 | LR |
| ipsi_G_temporal_inf_area | 0.9979 | LR |
| ipsi_G_temporal_middle_thickness | 0.4578 | LR&RF |
| ipsi_G&S_cingul-Ant_thickness | 0.9979 | LR |
| ipsi_G&S_paracentral_thickness | 0.9946 | RF |
| ipsi_S_cingul-Marginalis_area | 0.9979 | LR |
| ipsi_S_circular_insula_ant_thickness | 0.9946 | RF |
| ipsi_S_circular_insula_sup_thickness | 0.9979 | LR |
| ipsi_S_collat_transv_post_area | 0.4578 | LR&RF |
| ipsi_S_front_sup_thickness | 0.9979 | LR |
| ipsi_S_interm_prim-Jensen_area | 0.9979 | LR |
| ipsi_S_intrapariet&P_trans_area | 0.9979 | LR |
| ipsi_S_oc-temp_lat_area | 0.3459 | LR&RF |
| ipsi_S_oc-temp_med&Lingual_thickness | 0.1647 | RF |
| ipsi_S_pericallosal_thickness | 0.9946 | LR&RF |
| ipsi_S_temporal_sup_thickness | 0.4667 | RF |
| L-Sg_contra | 0.0512 | LR&RF |
| L-Sg_ipsi | 0.4885 | RF |
| MDm_contra | 0.7706 | RF |
| Pf_contra | 0.9655 | LR |
| RD-inferior_cerebellar_peduncle_contra | 0.9946 | RF |
| RD-sagittal_stratum_ipsi | 0.3404 | RF |

**Supplemental Table 5.** Top 50 most important predictors and univariate statistics for CTN vs TNP classification task. AD – axial diffusivity, FA – fractional anisotropy, HC – healthy controls, LR – logistic regression, RD – radial diffusivity, RF – Random Forest, TN – trigeminal neuralgia, TNP – trigeminal neuropathic pain.
